# Supplementary material for: Single cell transcriptome atlas of the Drosophila larval brain
Source: eLife. 2019 Nov 20;8:e50354. doi: 10.7554/eLife.50354 (PMC6894929; doi:10.7554/eLife.50354)
Supplement: Supplementary file 2. — Scatter plots illustrating the differentially expressed genes per cluster and per condition. Dark blue: a tendency line. Light-dashed line: FC=±1. [file elife-50354-supp2.pdf]

Scatter1

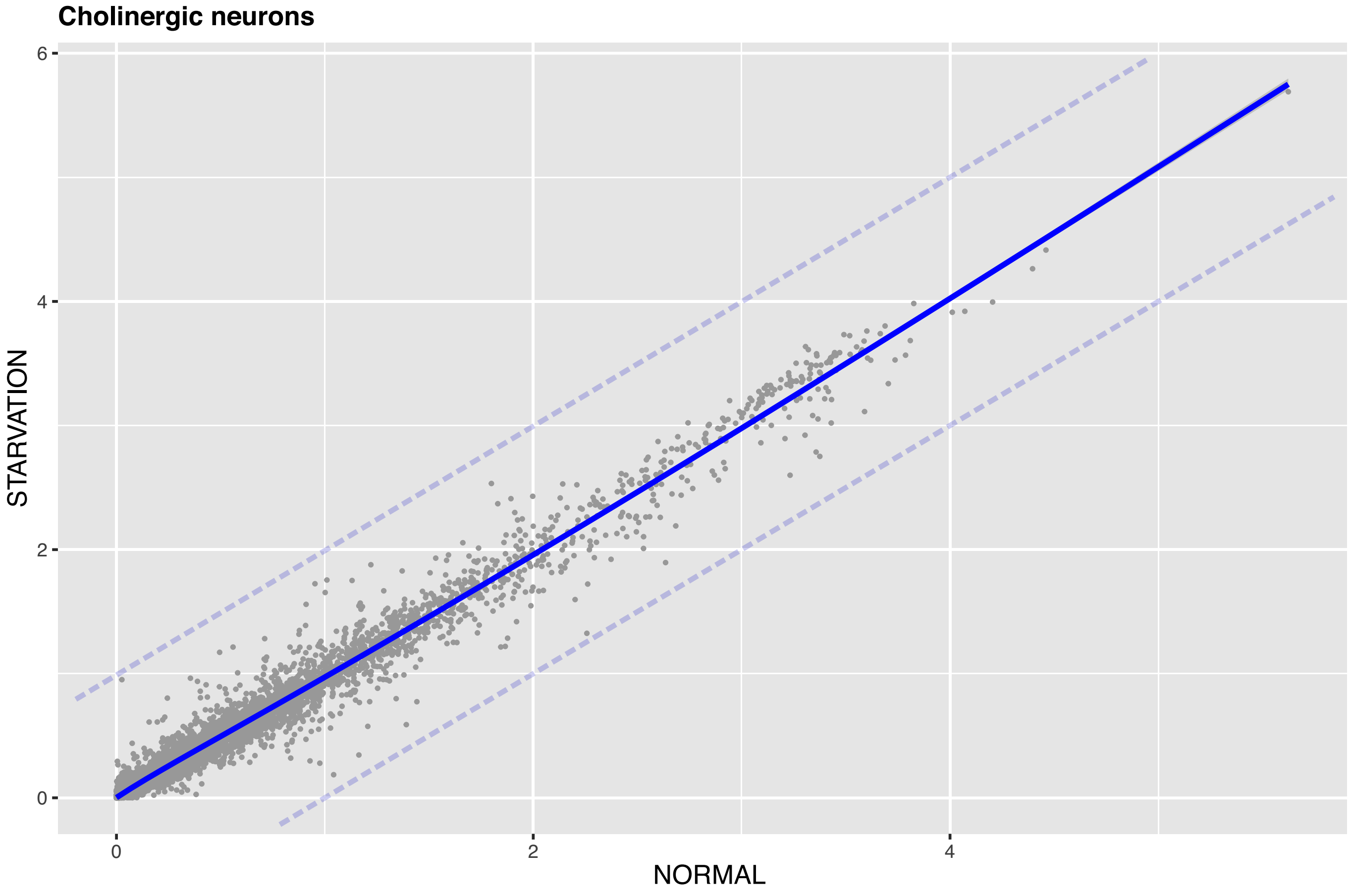
Cholinergic neurons
STARVATION
NORMAL
0
2
4
6
0
2
4

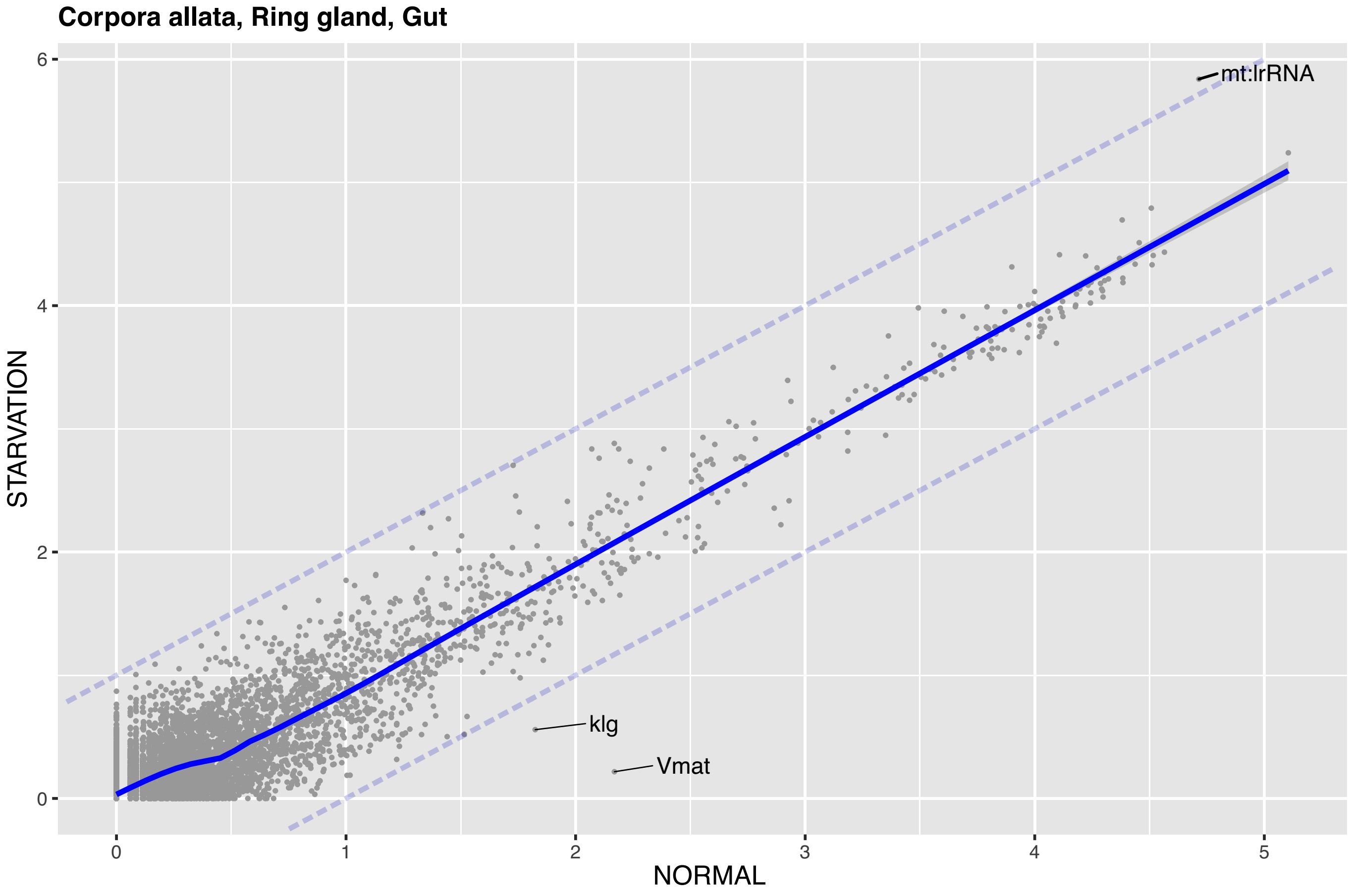
Corpora allata, Ring gland, Gut
STARVATION
NORMAL
mt:lrRNA
klg
Vmat
0
2
4
6
0
1
2
3
4
5

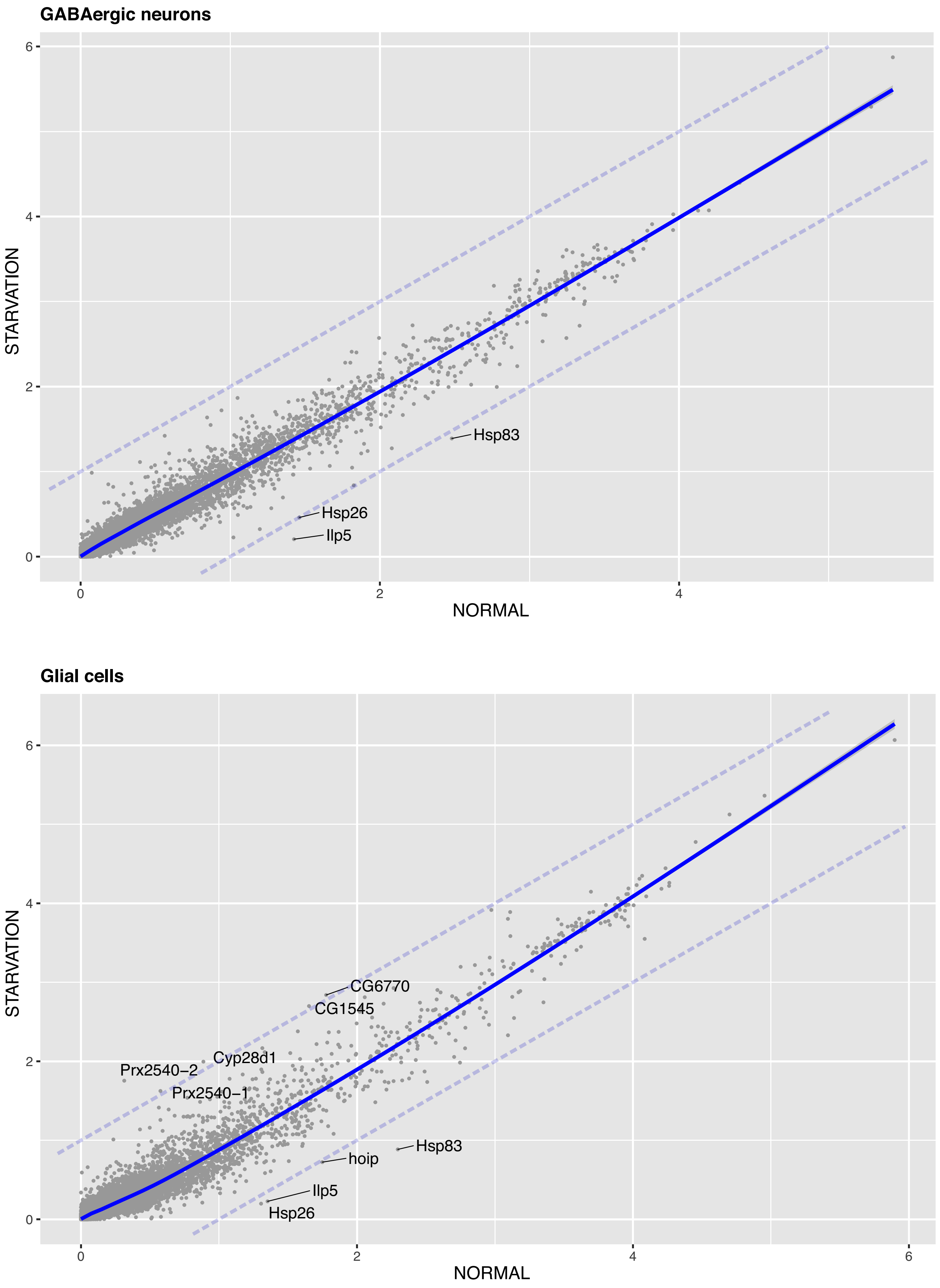
GABAergic neurons
STARVATION
NORMAL
0
2
4
6
Hsp83
Hsp26
Ilp5
Glial cells
STARVATION
NORMAL
0
2
4
6
CG6770
CG1545
Cyp28d1
Prx2540−2
Prx2540−1
Hsp83
hoip
Ilp5
Hsp26

Glutamatergic neurons

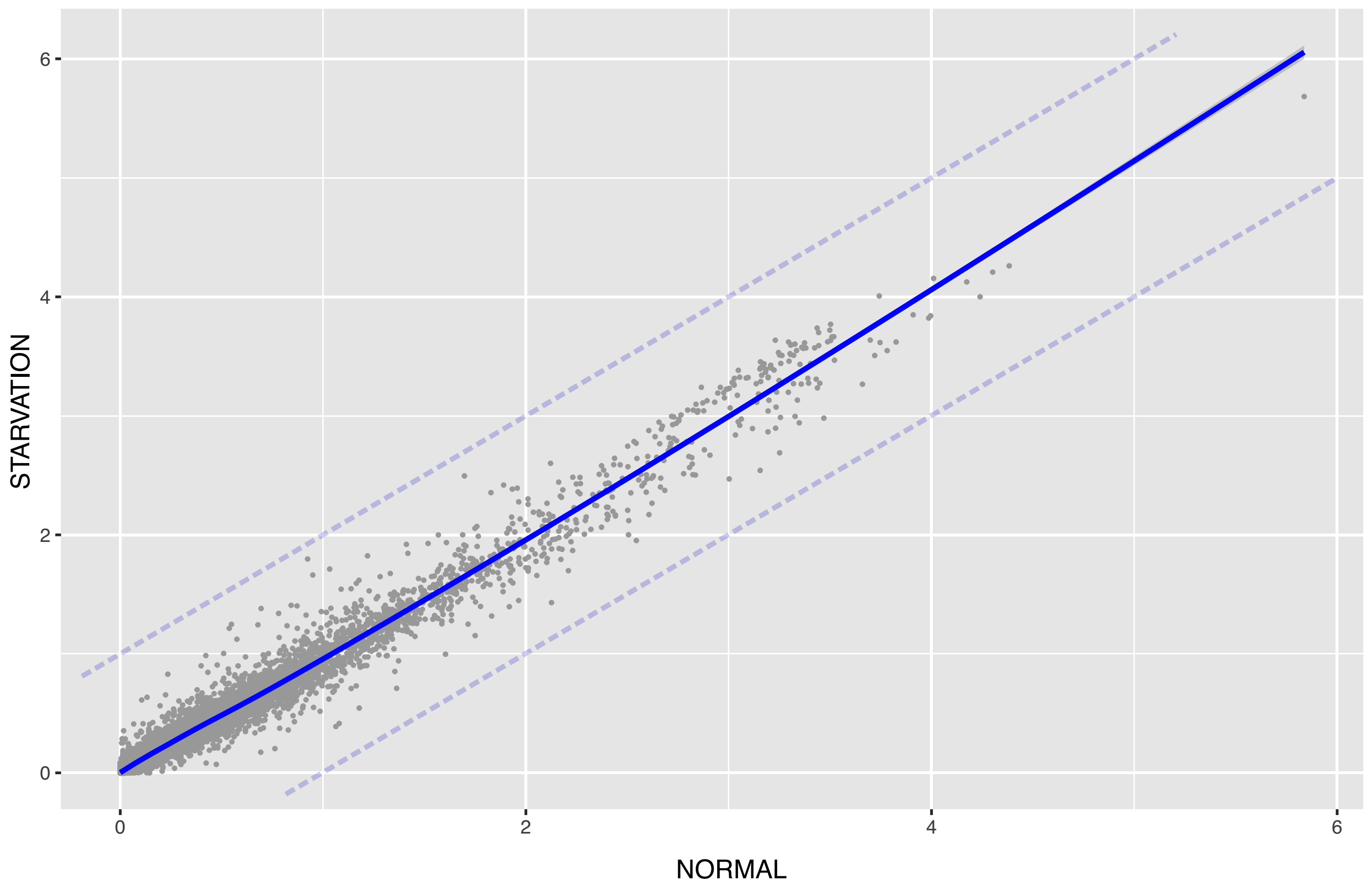

Hemocytes

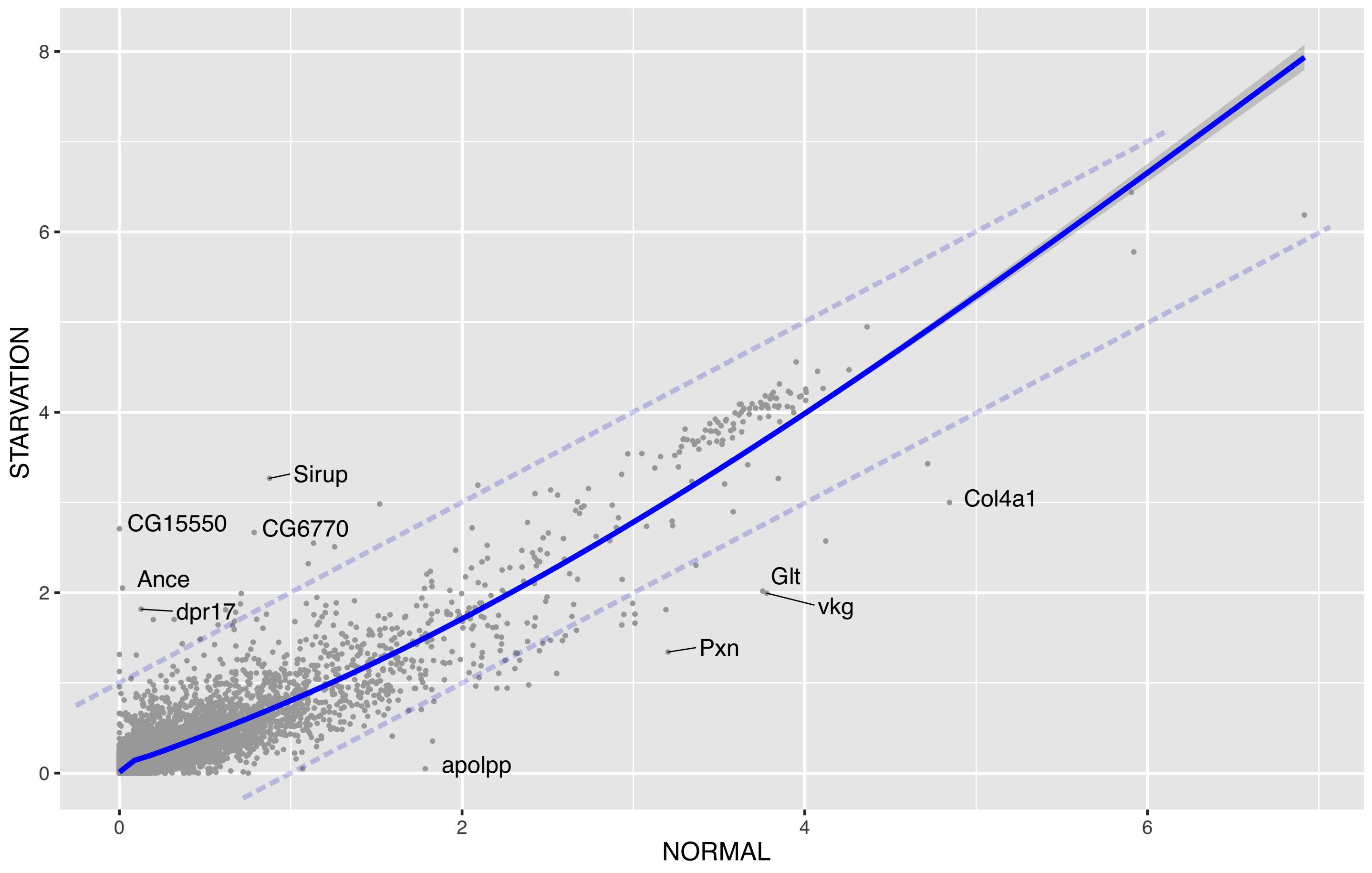

Monoaminergic neurons

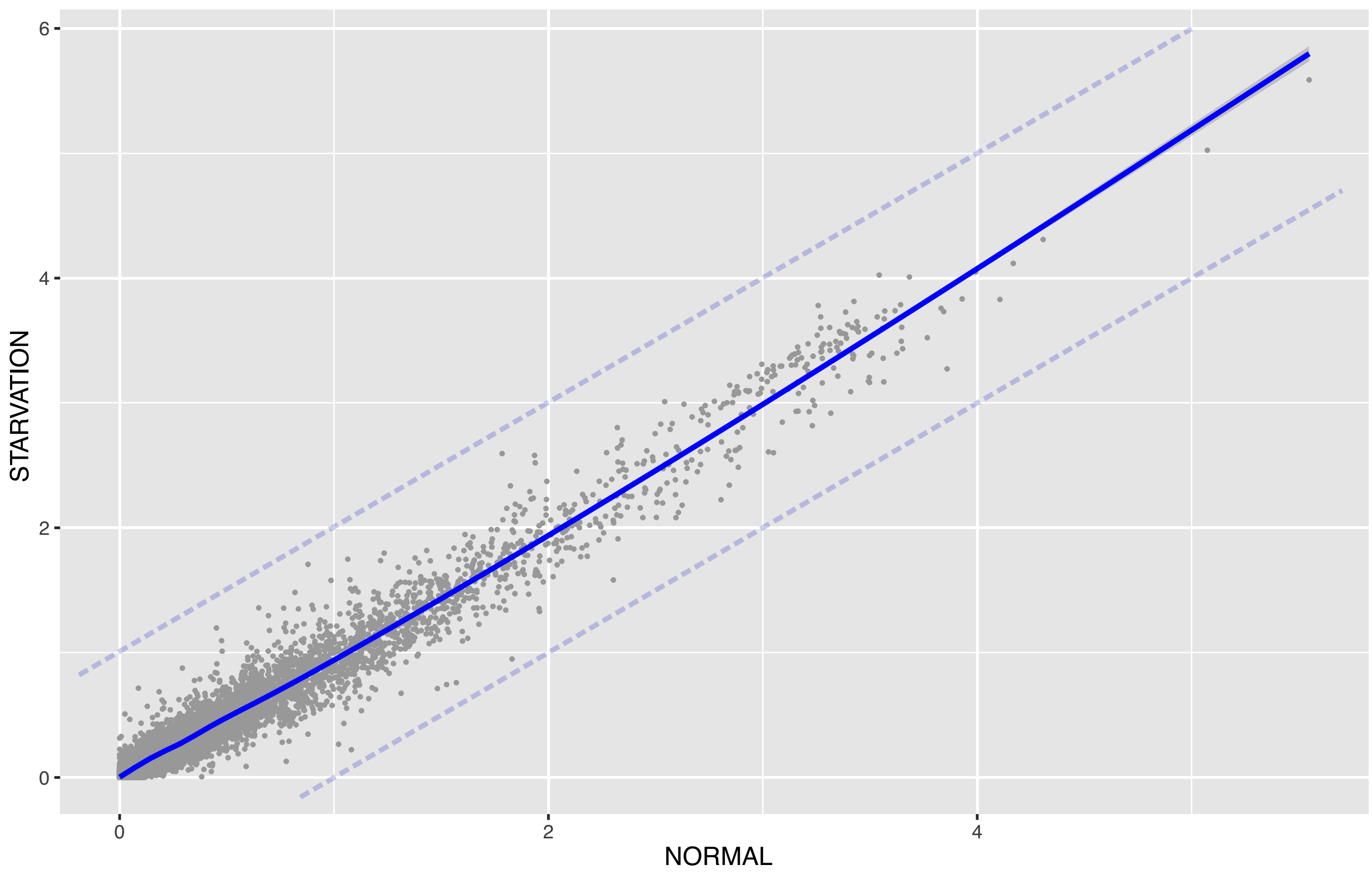

Muscle cells

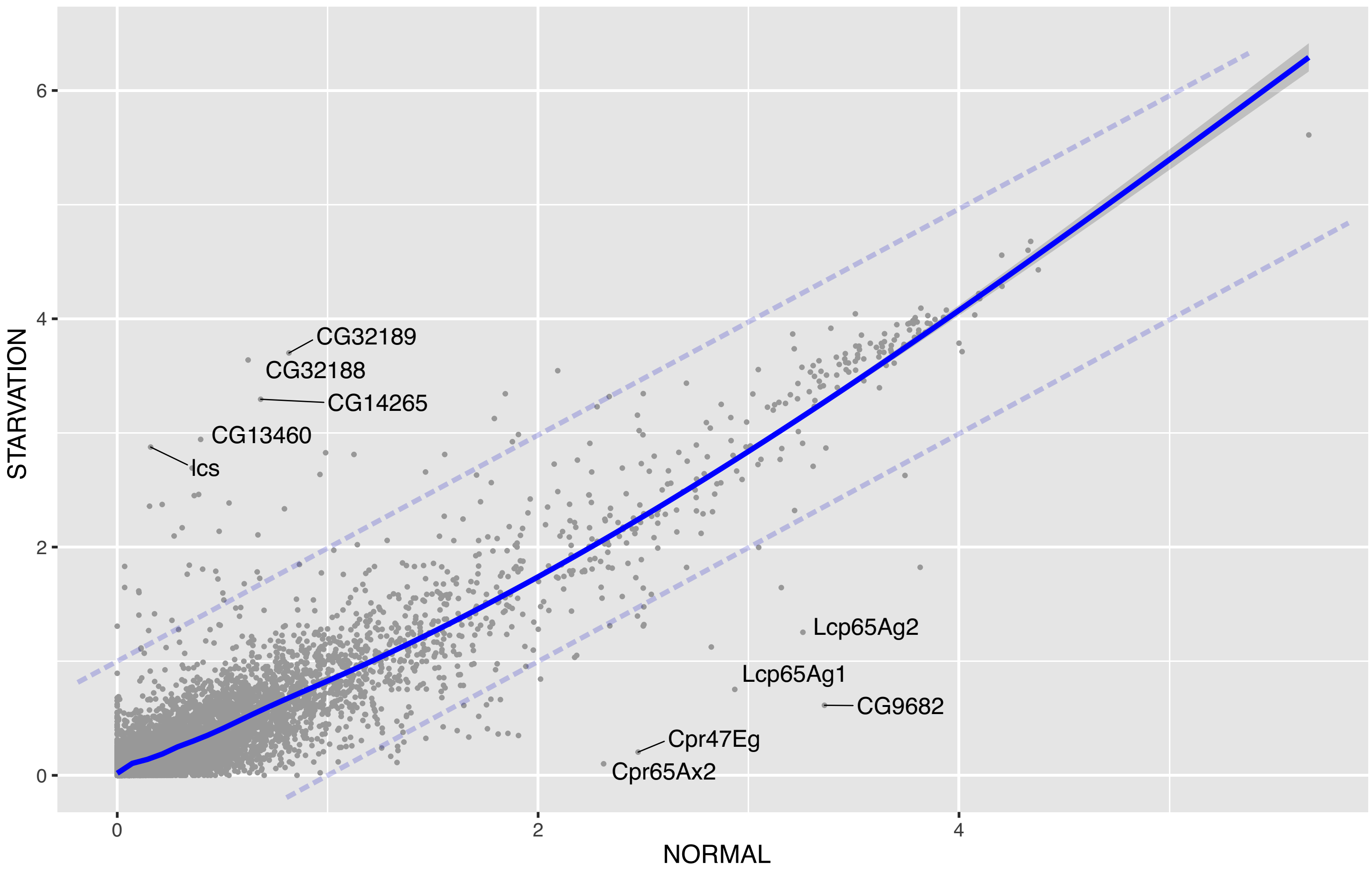

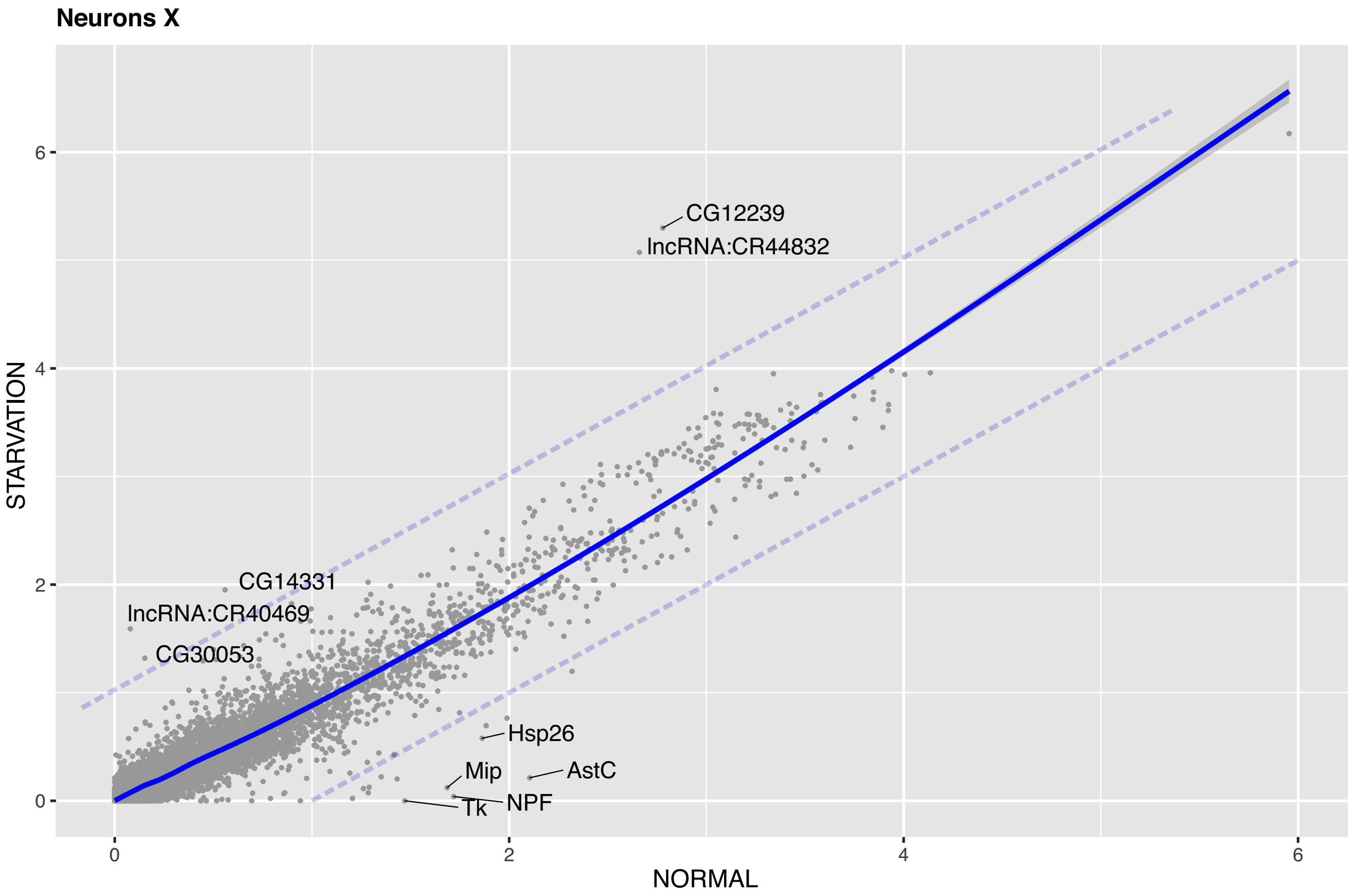
Neurons X
STARVATION
NORMAL
0
2
4
6
CG12239
lncRNA:CR44832
CG14331
lncRNA:CR40469
CG30053
Hsp26
Mip
AstC
Tk
NPF

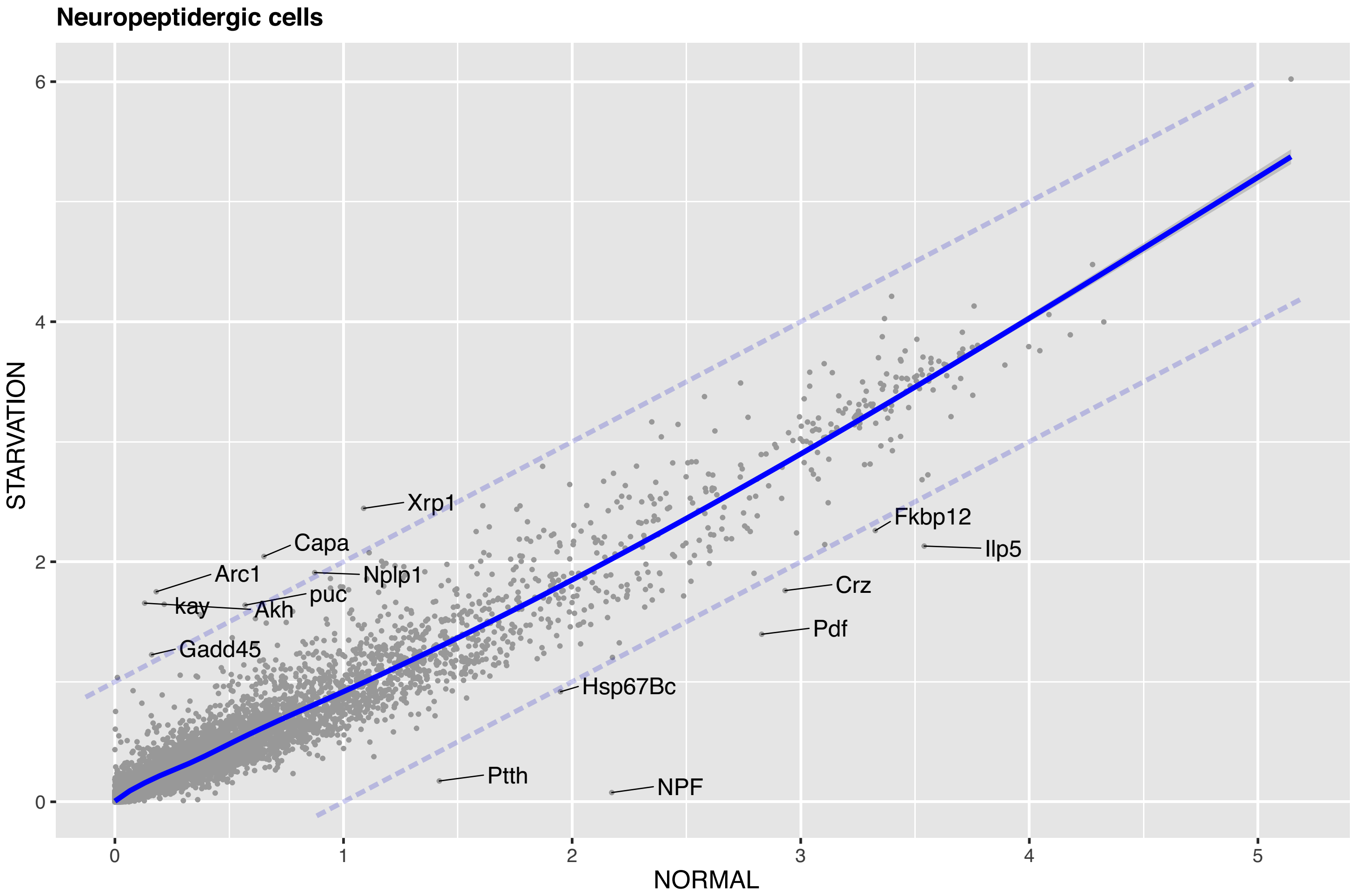
Neuropeptidergic cells
STARVATION
NORMAL
0
1
2
3
4
5
6
Xrp1
Capa
Arc1
Nplp1
puc
kay
Akh
Gadd45
Fkbp12
Ilp5
Crz
Pdf
Hsp67Bc
Ptth
NPF

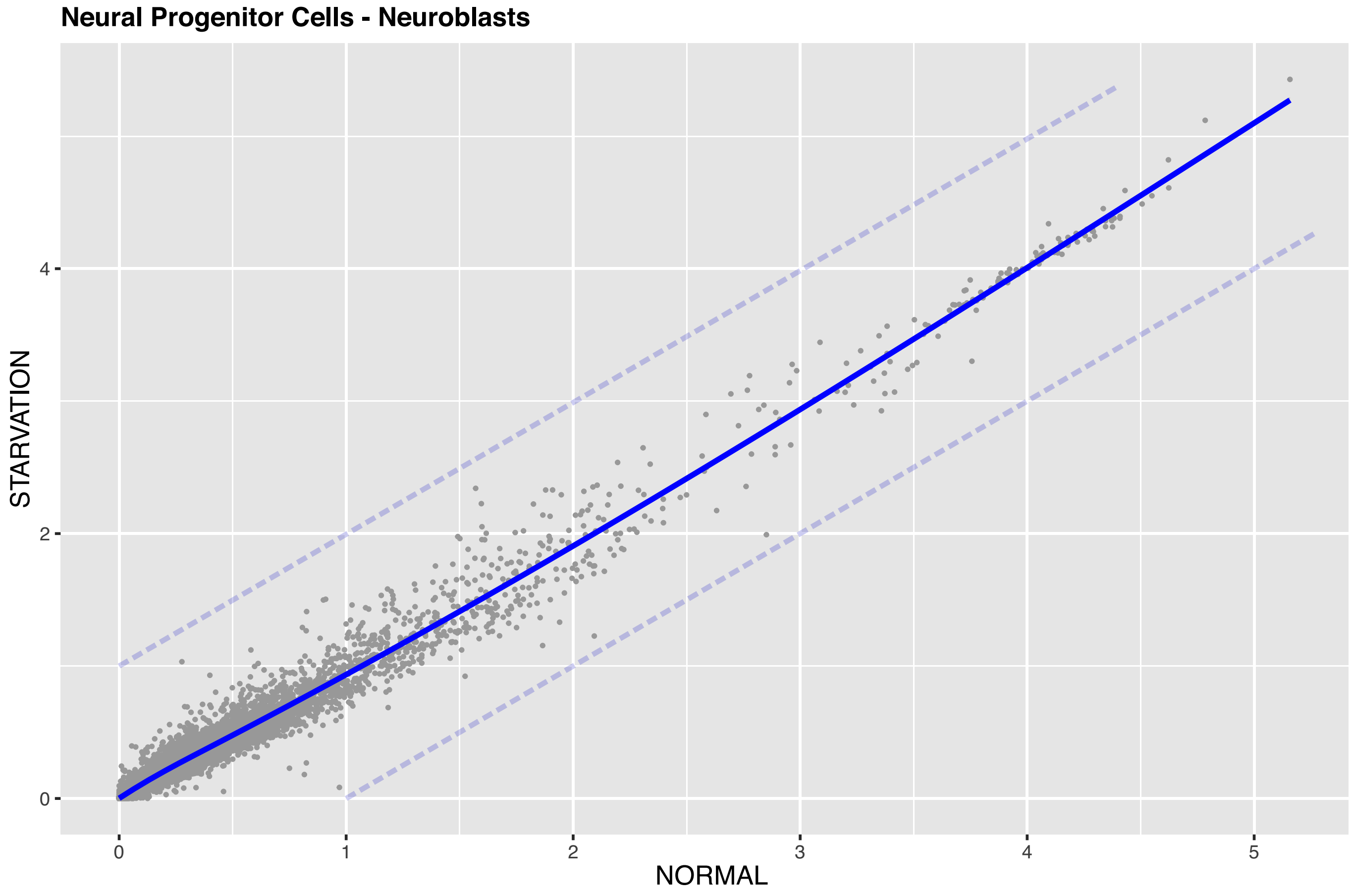
Neural Progenitor Cells - Neuroblasts
STARVATION
NORMAL
0
2
4
0
1
2
3
4
5

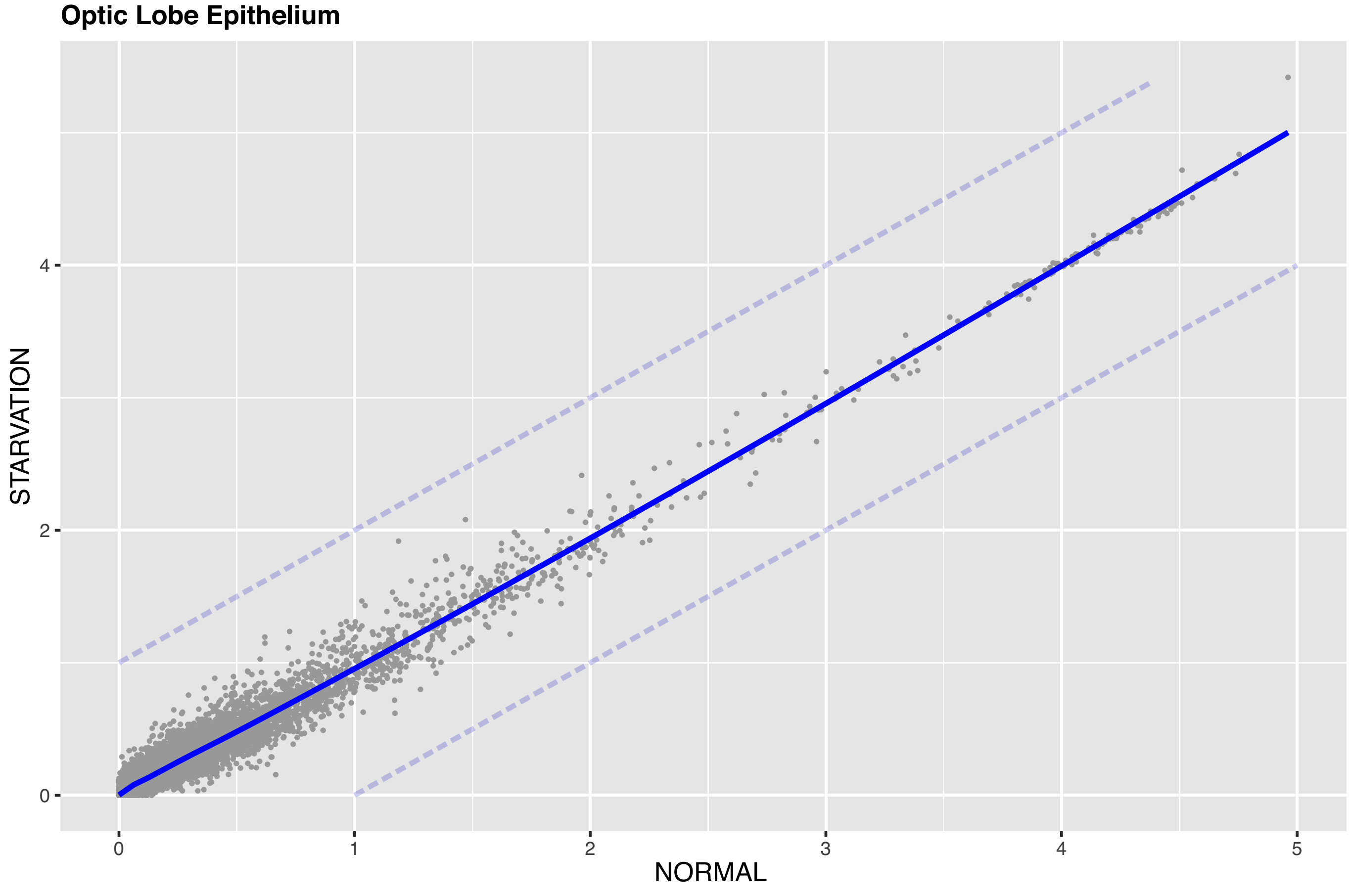
Optic Lobe Epithelium
STARVATION
NORMAL
0
2
4
0
1
2
3
4
5

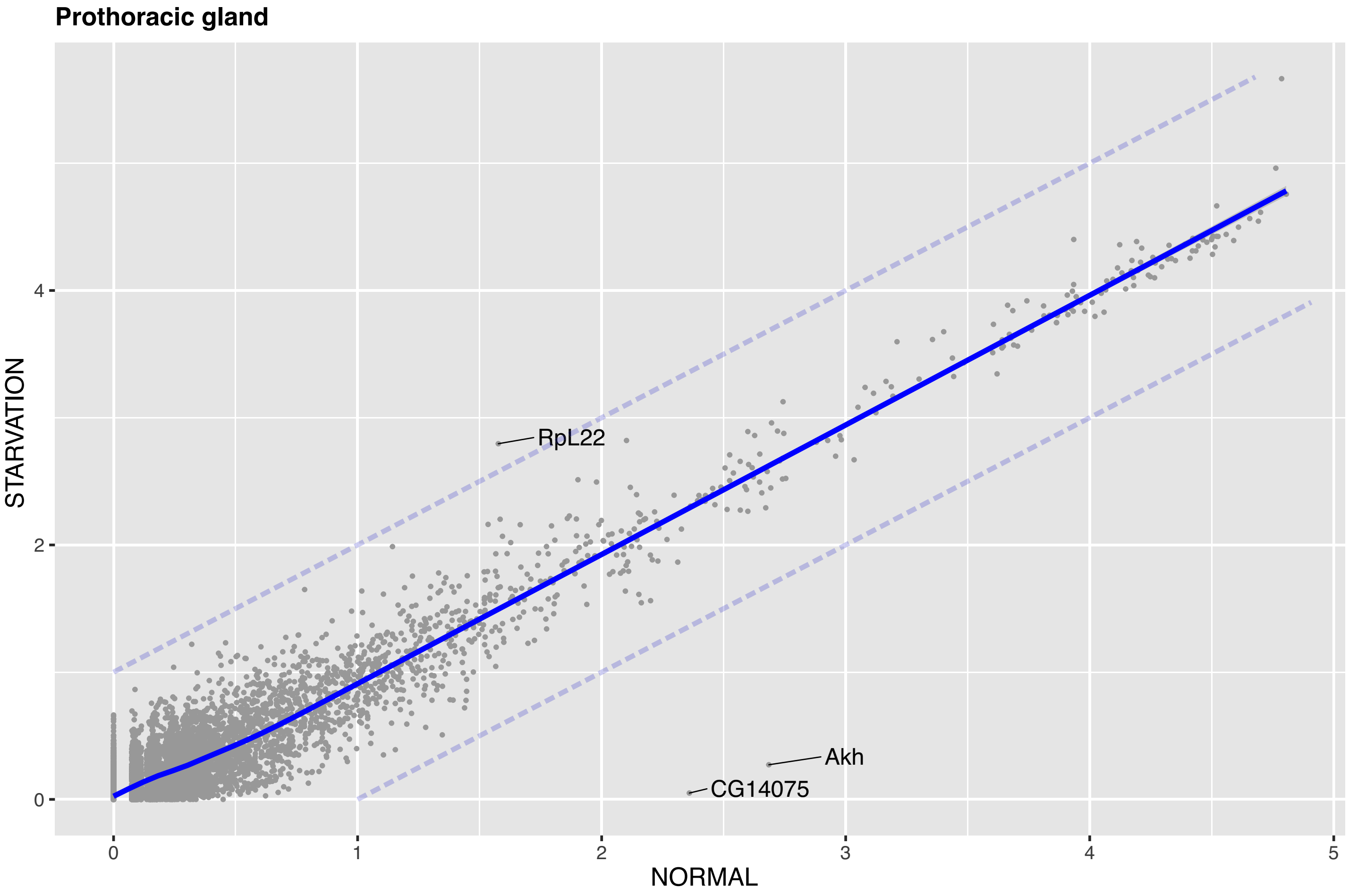
Prothoracic gland
STARVATION
NORMAL
0
2
4
0
1
2
3
4
5
RpL22
Akh
CG14075

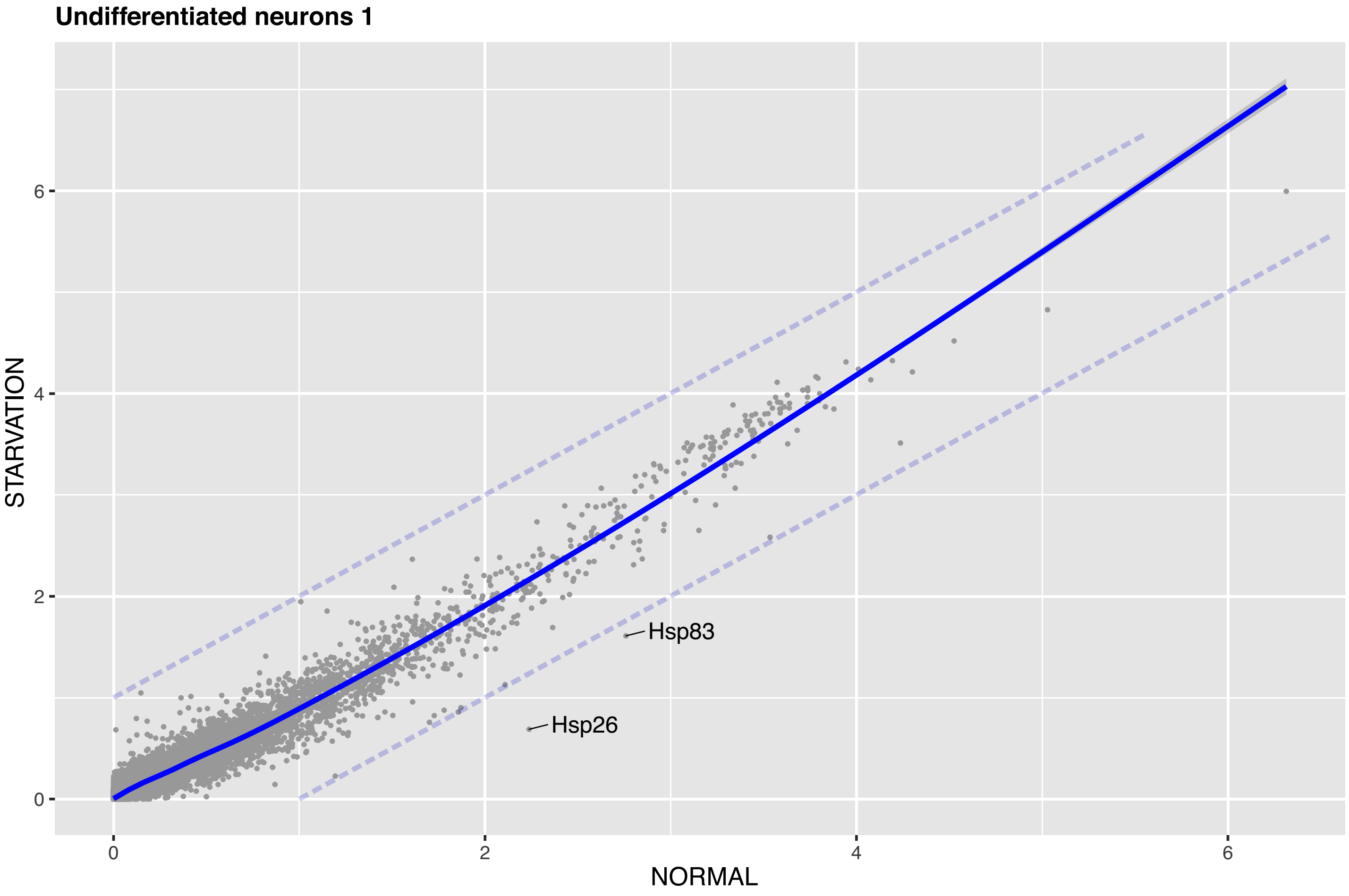
Undifferentiated neurons 1
STARVATION
NORMAL
0
2
4
6
0
2
4
6
Hsp83
Hsp26

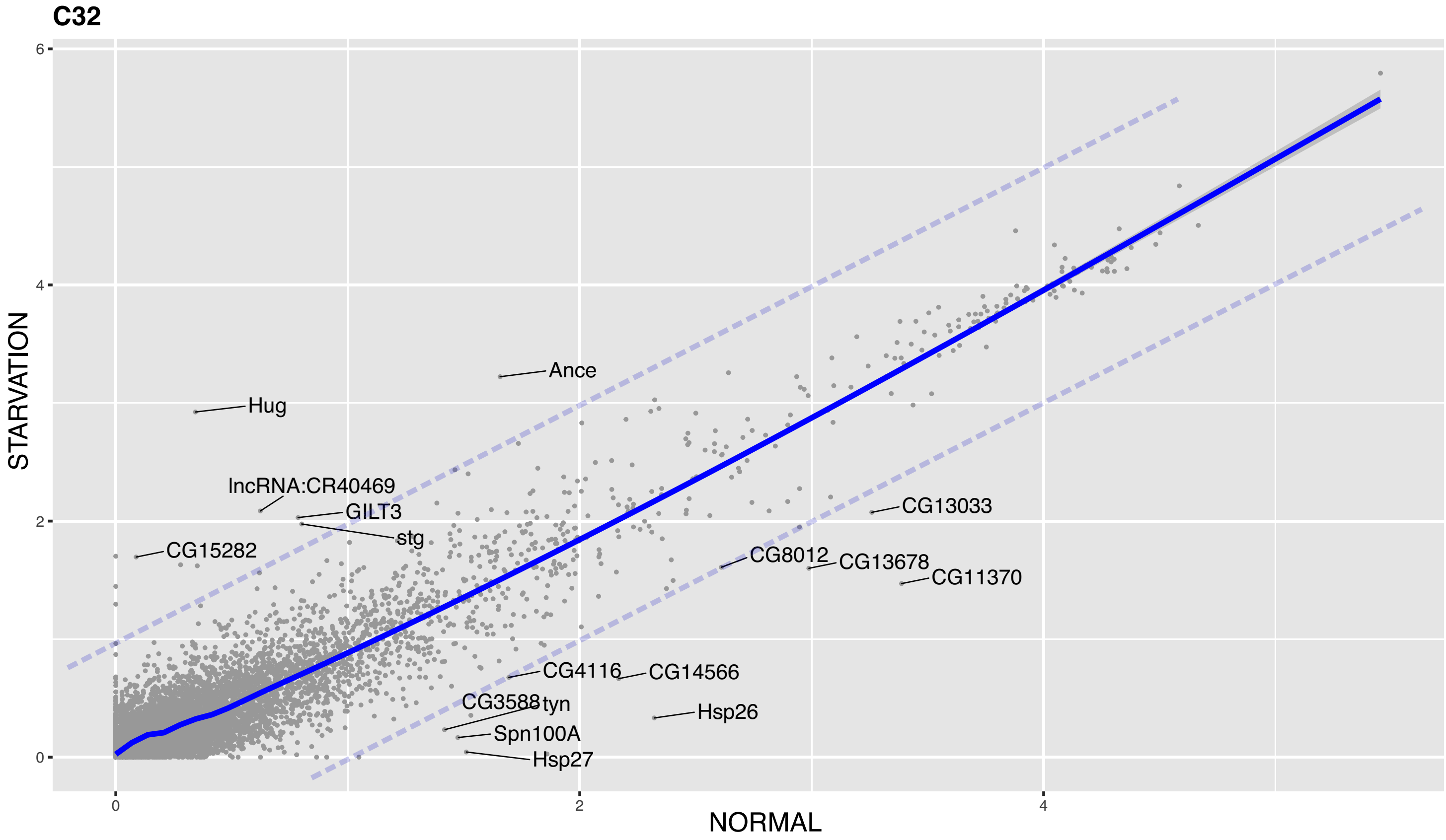

C32
STARVATION
NORMAL
0
2
4
6
0
2
4
Ance
Hug
lncRNA:CR40469
GILT3
stg
CG15282
CG13033
CG8012
CG13678
CG11370
CG4116
CG14566
CG3588
tyn
Hsp26
Spn100A
Hsp27
