# Supplementary figures and images for: Single cell transcriptome atlas of the Drosophila larval brain
Source: eLife. 2019 Nov 20;8:e50354. doi: 10.7554/eLife.50354 (PMC6894929; doi:10.7554/eLife.50354)

## Normal

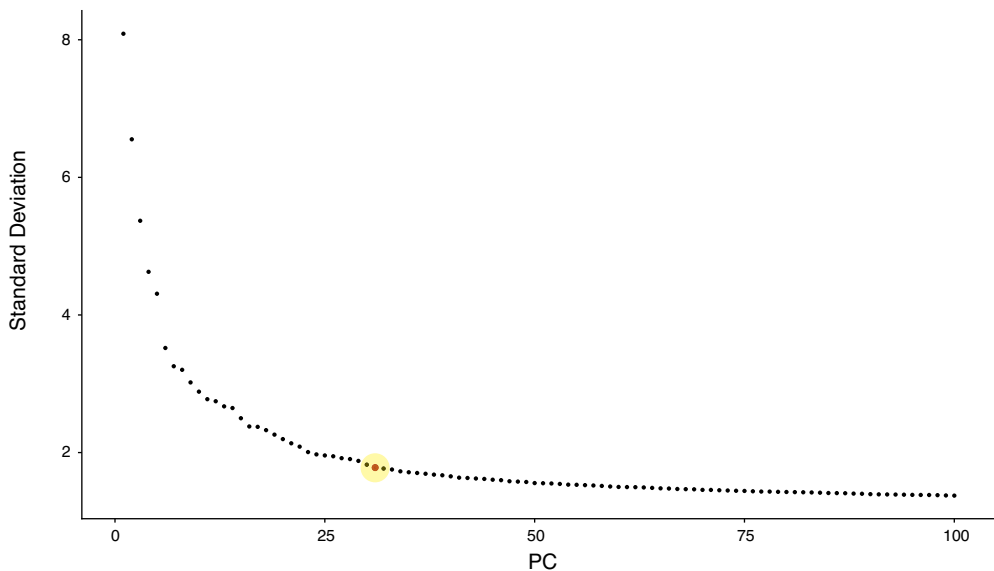

## Starvation

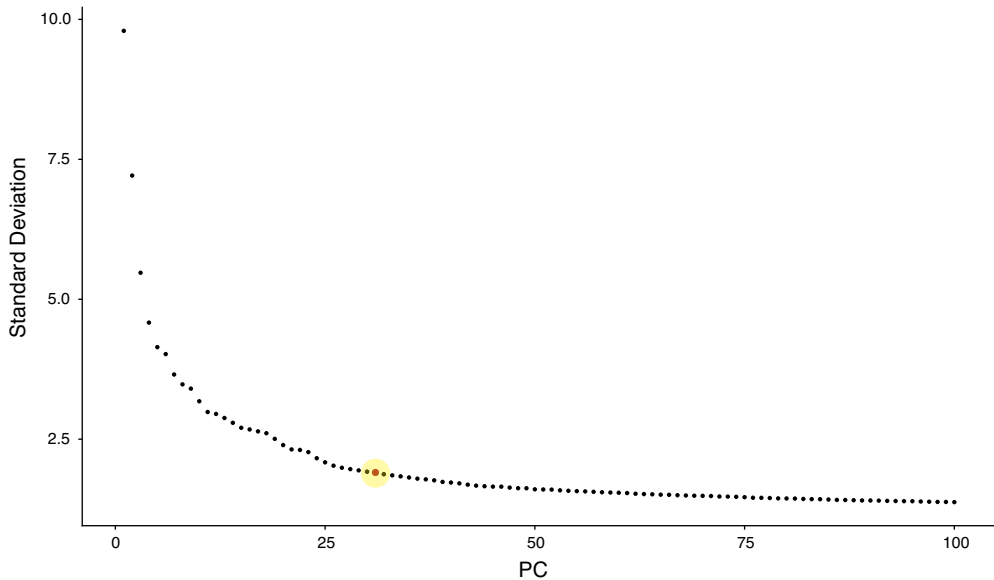

Supplement: Supplementary file 3. — Elbow plots analyzed to select the real dimensionality of the datasets. In red and pointed with an arrow, the number of PCs selected for downstream processing. [file elife-50354-supp3.pdf]
